# Biosensing of Urea with a Functionalized Gold Electrode for Health and Food Monitoring

**DOI:** 10.1021/acs.jafc.5c08426

**Published:** 2025-09-24

**Authors:** Angelo Ferlazzo, Meryam Chelly, Antonino Gulino, Giovanni Neri

**Affiliations:** † Department of Chemical Sciences and INSTM Research Unit, University of Catania, Viale Andrea Doria 6, 95125 Catania, Italy; ‡ Department of Engineering, University of Messina, Contrada Di Dio, 98166 Messina, Italy

**Keywords:** biosensor, urease, urea, milk, water, saliva

## Abstract

Urea monitoring in biological fluids is of crucial significance, since urea is a key indicator of liver and kidney physiological functioning, a marker for hemodynamic treatments, and has been used for food adulteration. Therefore, we have covalently anchored the urease enzyme (Ur) to a screen-printed gold electrode (SPGE) using the 3,3′-dithiodipropionic acid di­(*N*-hydroxysuccinimide ester) (DSP) bifunctional linker that allowed optimal electronic communication between urease and gold electrode and developed a selective urea biosensor (Ur-DSP/SPGE). The Ur-DSP/SPGE was characterized by infrared spectroscopy (FTIR), cyclovoltammetry (CV), and electrical impedance spectroscopy (EIS). Open-circuit potentiometry (OCP) was also used to detect urea at different concentrations (0–600 μM) in water. The biosensor showed a urea limit of detection (LOD) of 5.0 μM, as well as excellent temporal stability and selectivity. Urea sensing was also investigated in caw’s milk, human saliva, and tap water with excellent results and recoveries.

## Graphical abstract

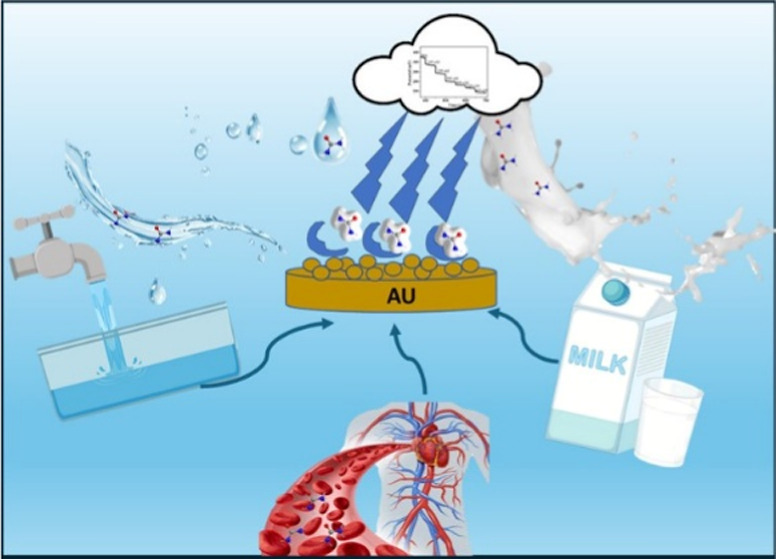

## Introduction

Biosensors in recent years have largely been investigated since they offer many advantages such as high sensitivity, speed, and ease of use and are suited for food analysis, environmental and health monitoring, etc.^1^

Urea is a key metabolite in the nitrogen cycle and a significant biomarker for assessing renal function, liver health, and protein metabolism and, therefore, is deeply measured in clinical studies.^2–5^ Its detection in biological fluids such as blood, saliva, and urine is useful to diagnose numerous diseases such as those involving kidney, heart, liver failure, and metabolic disorders.^6^ For example, the physiological presence of urea in urine must be within the 7–20 mg × dL^–1^ range (1.17–3.33 mM), and higher urea concentration values indicate kidney and/or liver diseases.^7,8^

Several studies have demonstrated a correlation between the amount of urea in blood and that found in saliva, and this is very important for the development of less invasive devices.^7,9^ In addition to the medical field, it is also of utmost importance to measure urea in food for quality control and pharmaceutical formulations or as an environmental pollutant.^3,10^ In fact, the addition of urea represents a food adulteration used to increase the nitrogen concentration that, usually, is correlated to the protein percentage. High levels of urea in water are very dangerous for human health, and its legal limit is 10 ppm (0.167 mM).^3^ Urea in milk should not exceed 70 mg × dL^–1^ (11.65 mM), and higher values indicate milk adulteration.^11–13^

There are many conventional analytical techniques for the determination of urea, including spectrophotometry,^14,15^ chromatography,^16^ fluorimetry,^17^ surface-enhanced Raman scattering,^18^ and enzymatic assays, and they provide high sensitivity but have also several limitations such as being unsuitable for on-site monitoring or requiring specialized personnel, therefore ill-suited for point-of-care or on-site analysis.^19^

Many of these limitations can be overcome using enzyme-based biosensors to monitor urea, and urease allows the catalytic urea degradation in particular reaction conditions.^20–22^ Some optical urea biosensors based on a nanozyme, Au@urease NPs, carbon-dots-Ag NPs, and bilayer actuator films have been proposed, but the obtained limits of detection are not so exciting.^23–26^

In this complex field, electrochemical biosensors, particularly those employing potentiometric detection schemes, offer an attractive alternative due to their low power consumption, fast response, ease of miniaturization, and potential for real-time analysis. Among these, enzyme-based biosensors utilizing urease, the enzyme that catalyzes the hydrolysis of urea into ammonia and carbon dioxide, are widely used.^27,28^ However, the stability, reproducibility, and sensitivity of urease-based biosensors are often limited by the method of enzyme immobilization on the electrode surface. One of the key steps in the fabrication of electrochemical enzyme-based biosensors is the enzyme immobilization on the electrode that can affect the biosensor stability and result’s repeatability.^21,29^ Several methods are reported in the literature for the enzyme immobilization, which often involve the use of different polymers such as polyaniline, polypyrrole, poly­(*o*-phenylenediamine), chitosan, and poly­(vinyl chloride), either by entrapment of the enzyme directly during electropolymerization or by physical adsorption and chemical cross-linking.^30^ Enzyme adsorption suffers from inevitable enzyme leaking or desorption due to the presence of the enzyme at the film/solution interface. Other techniques, such as cross-linking and polymer addition after enzyme adsorption, have the advantage of improving the stability of the enzyme in the polymer film, but sensitivity is often affected by the increasing difficulty of accessibility to the enzyme active site.^31^

It is therefore of fundamental importance to develop stable sensors that can quickly and accurately be used to monitor urea, to check the health status of humans and detect the presence of adulterations in food, and to monitor environmental pollution of water.

Covalent immobilization strategies have emerged as a robust solution to improve the enzyme retention and operational stability. In particular, bifunctional linkers can provide controlled and stable enzyme attachment while preserving catalytic activity. Nevertheless, efficient and straightforward chemistries enabling such covalent binding, especially on gold substrates, remain underexplored.

Therefore, in this work, we report a novel and simple method to covalently immobilize urease onto screen-printed gold electrodes (SPGE) using 3,3′-dithiodipropionic acid di­(*N*-hydroxysuccinimide) ester (DSP), a bifunctional cross-linker that allowed easy fabrication of an enzyme potentiometric urea sensor. DSP enables stable thiol-gold anchoring and efficient enzyme coupling via amine-reactive NHS esters through the formation of an amide bond. This covalent interface forms the basis of a potentiometric urea biosensor with high sensitivity and selectivity. This biosensor exhibited excellent performance in detecting micromolar concentrations of urea, even in complex real matrices such as milk, saliva, and environmental water samples.

In this context, it is worthy of note that SPEs, which use thick-film technology, have widely been used for sensor development. This technology allows for the production of solid strip electrodes that are reproducible, inexpensive, and mechanically robust. SPE has many advantages such as small size, low cost, ease of use, fast response, and the possibility of using different inks (carbon-, silver-, gold-based nanomaterials, etc.). Thanks to these peculiarities, SPEs can be used as transducers in a wide variety of biosensors to develop innovative and portable electrochemical sensing platforms.^32,33^ In fact, it has recently been developed the first electrochemical sensor, based on an array of hollow MgCl_2_ microneedles deposited on SPEs, useful to simultaneously detect urea and pH in a interstitial fluid matrix.^34^ Also, Ag NPs anchored on nitrogen-doped graphene nanoplatelets were deposited on SPEs for amperometry detection of urea.^35^ Likewise, a new impedance electrochemical sensor for urea detection was obtained with SPEs modified with CuO/Co_3_O_4_@MWCNTs.^36^

Therefore, it emerges that modified SPEs could be the future for urea electrochemical biosensing, and this study represents the first report on the use of DSP as a covalent anchoring agent that allowed optimal electronic communication between urease and gold electrodes (vide infra). This new Ur-DSP/SPGE biosensor showed a very low LOD, good stability, and high repeatability of the results. The Ur-DSP/SPGE biosensor was also used to detect urea in milk, saliva, and water real samples. To the best of our knowledge, this is the first report of covalent urease immobilization on gold electrodes using DSP as a bifunctional linker. Our results demonstrate that this strategy provides a stable and reproducible sensing platform with significant potential for clinical, food safety, and environmental monitoring applications.

## Materials and Methods

### Instruments and Chemicals

Urea (powder, purity ≥99.0%), phosphate buffered saline (PBS) tablets, and urease from *Canavalia ensiformis* (Type C-3, powder, 600,000 units/g solid) were obtained from Sigma-Aldrich; 3,3-dithiodipropionic acid di­(*N*-hydroxysuccinimide) ester powder was obtained from Merck Life Science S.r.l. The IR study of the sensor modification with the urease enzyme was conducted by using a Perkin Spectrum 100 FTIR spectrometer, equipped with a universal ATR sampling accessory. FTIR analyses were performed at RT in the 4000–400 cm^–1^ range and with a 4.0 cm^–1^ resolution. pH measurements were performed with a Metrohm 913 pH meter 913.

### Fabrication of the Ur-DSP/SPGE Biosensor

Figure 1 shows the synthetic scheme for the Ur-DSP/SPGE biosensor fabrication. Ten microliters of a DSP solution in DMSO (10 mg/mL, 2.47 × 10^–5^ mol/L) were deposited via drop casting on the gold working electrode of a SPGE and left 2 days to dry at room temperature. Then, 10 UI of the urease enzyme (10 μL of a solution in PBS containing 1.6667 × 10^–2^ g of urease) was deposited on the functionalized gold working electrode, at 0 °C (24 h). The immobilization of urease on DSP was achieved by the formation of an amide bond between the –NH_2_ group of the enzyme and the −COO– group of the linker, anchored to SPGE by an S–Au bond. After each step, the electrode was washed tree times with 5 mL of a 0.01 M PBS water solution to remove excess of DSP or enzyme from the sensor surface. Then, the Ur-DSP/SPGE was left at 0 °C for 1 day in a refrigerator.

**Figure 1:**
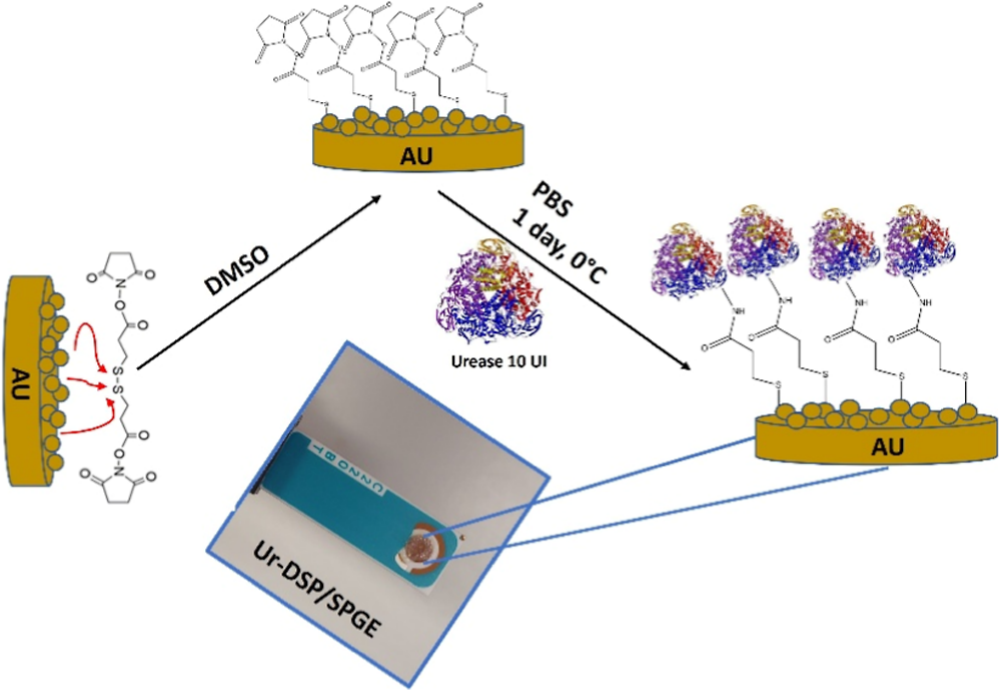
Schematic representation of the Ur-ΔSP/SPGE biosensor fabrication.

### Electrochemical Measurements

Electrochemical analyses were conducted using a DropSens μStat 400 potentiostat (DropSens, Spain), powered by Dropview 8400 data acquisition software, for CV and OCP analyses; a Metrohm Autolab Galvanostat potentiostat was used for EIS analyses.^37^ CV analyses were conducted in 0.01 M PBS (pH 7.4) in the −0.3–1.0 V potential range, in the presence of 10 mM ferri/ferrocyanide ([Fe­(CN)_6_]^4–/3–^). OCP tests were conducted by monitoring the variation of the potential over time in 0.01 M PBS, depending on the urea concentration. EIS analyses were conducted in a 0.1–105.0 Hz frequency range, amplitude 5 mV, applied potential 0.25 V, using a solution of 10 mM [Fe­(CN)_6_]^4–/3–^ and 0.1 M KCl. Electrochemical measurements were performed using screen-printed gold electrodes purchased from Metrohm-DropSens (Metrohm Italiana S.r.l., Origgio (VA), Italy). The SPGE consists of a planar substrate with both gold auxiliary and working electrodes (4 mm diameter and 0.1257 cm^2^ geometric area), while the reference electrode is Ag/AgCl. The LOD was calculated by multiplying by 3.3 the ratio between the standard error of intercept and the slope of the calibration line.^38^

### Real Sample Analyses

The typical physiological urea concentration in biological fluids ranges from 1 to 8 mM (2.5–7.5 mM in the blood; ∼3 mM in both saliva and milk). Therefore, saliva and milk samples for analyses were diluted using a 0.01 M PBS aqueous solution to get a 1:40 ratio.^39^ In addition, diluted saliva and milk samples were further analyzed after the addition of 80 μL of a 10 mM urea in PBS water solution (8 × 10^–7^ mol, 200 μM), thus resulting in 0.201–0.203 mM final urea solutions (total volume = 4.08 mL). Tap water was analyzed before and after the addition of 16 μL of a 10 mM urea in PBS water solution (1.6 × 10^–7^ mol), thus resulting in a 40 μM final urea solution (total volume = 4.016 mL). We performed OCP measurements to quantify the total urea concentration in the above-mentioned real samples.^40^ Considering the dilution, we investigated urea concentrations in the starting samples up to 24 mM. To validate the results obtained using the present Ur-DSP/SPGE sensor, we also measured the urea concentration using the NADH method by means of a urea Assay Kit III provided by Sigma-Aldrich.

A measure of a given analyte amount (urea in our case), probed by a given analytical method, with respect to the total real analyte quantity, is defined as Recovery and can be calculated according to eq 1:

1 $$recovery\;\left(\%\right)=\frac{R_{U}-R_{0}}{RS_{U}}\times 100$$

where *R*_u_ and *R*_0_ are the analytical responses of the probe in real samples in which urea was added, and blank, respectively, and *R*_S_ denotes the analytical response of an equivalent concentration of urea in a PBS solution.^41^ The recovery values can sometimes exceed 100%.^42^

## Results and Discussion

### FTIR Characterization of Ur-DSP/SPGE

Covalent anchoring of the enzyme is the most advanced technique for immobilizing it on the surface of the working electrode, and this not only inhibits leaching of the enzyme but also provides an enzyme monolayer, which increases sensitivity and long-life stability of the biosensor. Functionalization of the gold working electrode was followed by FTIR.^43^ Figure 2 shows the FTIR spectra of bare SPGE, DSP/SPGE, urease, and covalently functionalized (with the urease enzyme) Ur-DSP/SPGE sensors. The SPGE sensor shows only a weak broad band at 3300–3500 cm^–1^ due to the O–H stretching of water on the electrode surface (see Table S1). In contrast, the DSP/SPGE sensor shows the absorption band at 1712 cm^–1^, attributable to the C=O group, and the presence of a band at 1020 cm^–1^ attributable to the N–C–O group, characteristic of the DSP molecule.^44^ Furthermore, the absence of any band at ∼2552 cm^–1^, characteristic of free S–H groups,^45^ confirms the functionalization of the gold electrode with DSP, by the formation of S–Au bonds. The FTIR of the pure urease enzyme shows a weak band at 1648 cm^–1^ related to a protein peptide bond and a more intense band at 1140 cm^–1^ attributable to the C–O of the carboxylic acid.^29,46^

**Figure 2:**
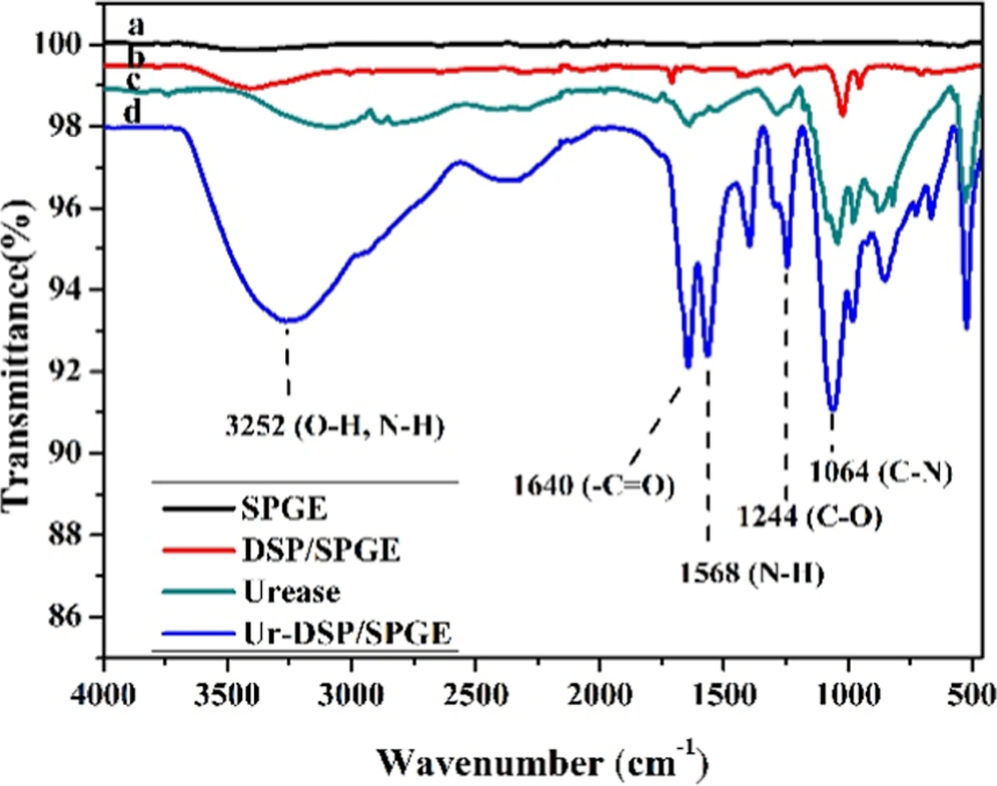
FTIR analyses of SPGE (line a), DSP/SPGE (line b), urease enzyme (line c), and Ur-DSP/SPGE biosensor (line d).

The FTIR spectrum of the Ur-DSP/SPGE sensor shows the presence of numerous absorption bands. In particular, the strong broad band at 3252 cm^–1^ indicates the presence of numerous O–H and NH_2_ groups already present in the structure of the enzyme; the intense band at 1640 cm^–1^, slightly shifted with respect to the urease enzyme, is attributable to the stretching of the C=O of the amide, formed between the DSP and the enzyme; and the band at 1568 cm^–1^ indicates the bending of the N–H group.^46,47^ As a whole, the FTIR bands above strongly indicate that the urease was covalently grafted on the DSP/SPGE sensor surface.

### Electrochemical Characteristics of Ur-DSP/SPGE

The electrochemical characteristics of the biosensors were investigated by CV and EIS analyses (Figure 3). Both functionalized DSP/SPGE and Ur-DSP/SPGE sensors show a decrease in peak intensity (*I*_pa_) and a significant Δ*V* increase (Δ*V* = 340 and 460 mV, respectively, Figure 3a) with respect to the simple SPGE. The Δ*V* increase indicates a sensor decreased conductivity due to the electrode passivation caused by the functionalization.^48,49^

**Figure 3:**
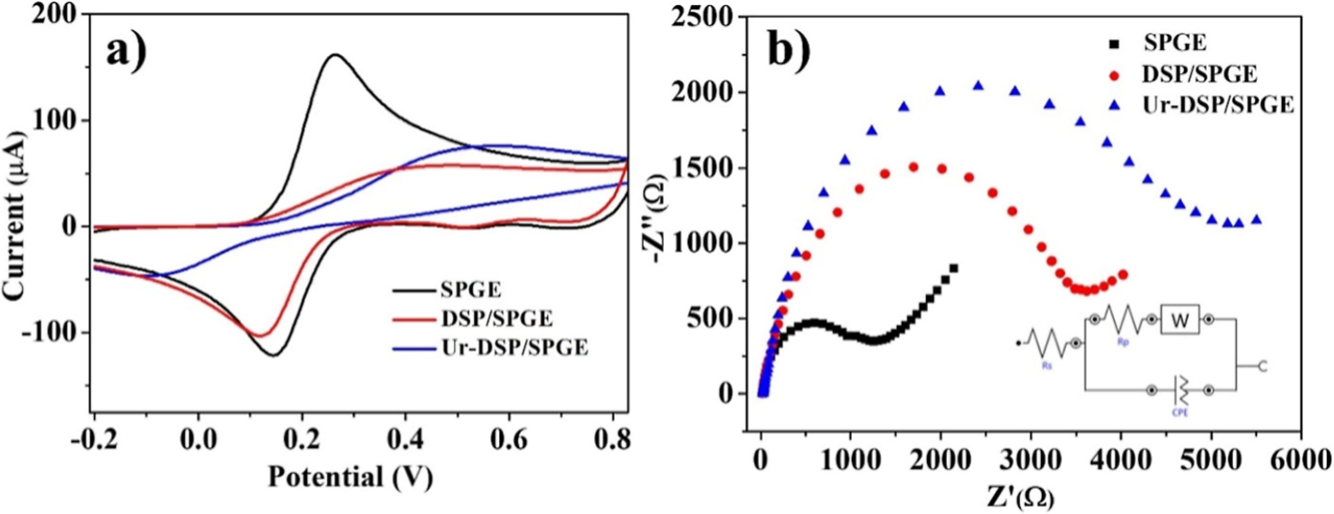
(a) CV for sensors immersed in a 10 mM [Fe­(CN)_6_]^4–/3–^ and 0.01 M PBS solution, at a scan rate of 50 mV/s. (b) EIS of sensors immersed in a 10 mM [Fe­(CN)_6_]^4–/3–^, 0.01 M PBS, and 0.1 M KCl solution, in a 0.1–105 Hz frequency range, amplitude 5 mV; inset shows the equivalent circuit.

Figure 3b shows the Nyquist diagram, obtained using EIS, where the *X* axis shows the real impedance and the *Y* axis shows the imaginary impedance. In particular, the *X* axis represents three components: the electrolytic resistance between the working and reference electrodes (*R*_s_), the double-layer capacitance (*C*_dl_), and the charge transfer resistors (*R*_ct_). Furthermore, the diffusion of molecules or redox species can create an additional frequency-dependent resistance, as at low frequencies, redox molecules can diffuse and increase the so-called Warburg (W) resistance.^50^ The values of the electronic transfer resistance (*R*_ct_ or *R*_p_) were 1406, 3668, and 5064 Ω for SPGE, DSP/SPGE, and Ur-DSP/SPGE, respectively.^51^ The observed increase in *R*_ct_ is related to the decrease of the electrode charge transfer capacity due to the functionalization.^48^

### Potentiometric Detection of Urea with Ur-DSP/SPGE

The ability of the Ur-DSP/SPGE sensor to detect different urea concentrations was investigated using OCP. Compared to other electrochemical techniques, potentiometry displays many advantages, such as high sensitivity, easy operation, and a simple apparatus.^52^

Urease is an enzyme that catalyzes the conversion of urea to NH_3_ and CO_2_, which then dissociates to NH_4_^+^ and bicarbonate HCO_3_^–^ ions in solution^21,53^

$${CH}_{4}N_{2}O+{3H}_{2}O+urease\to {2NH}_{4}^{+}+{HCO}_{3}^{-}+{OH}^{-}$$

Therefore, the potentiometric detection of urea can be performed since NH_4_^+^ ions generate a negative potential variation proportional to the NH_4_^+^ activity in buffer solutions.^24,53,54^

The observed potential variation for different urea concentrations is shown in Figure 4a,and Figure 4b shows the calibration curve, where the potential difference, with respect to the starting PBS solution, was plotted against pUrea. Note that the PBS addition does not produce any potentiometric variation (Figure S1). Based on the slope of the dynamic range, the Ur-DSP/SPGE sensor displays good sensitivity (about 3.92 mV μM^–1^ cm^–2^) to urea in water solution.^55^

**Figure 4:**
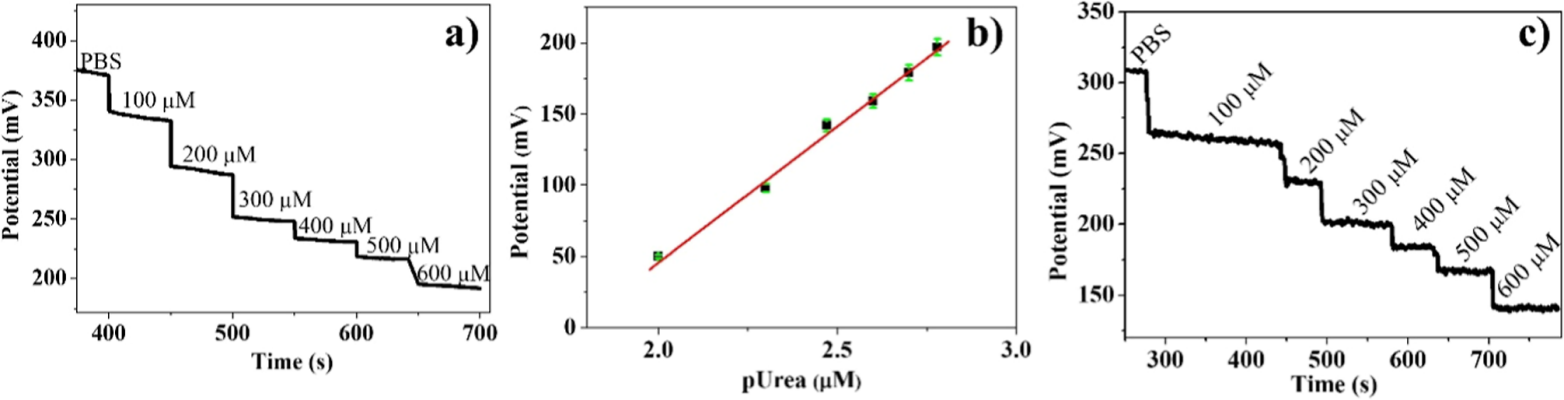
(a) Potentiometric response of the Ur-DSP/SPGE sensor versus time in the 0–600 μM urea range. (b) Calibration line of the Ur-DSP/SPGE sensor (*y* = *a* + *bx*; *R* ^2^ = 0.99289; slope = 189.207 ± 7.15496). (c) Potentiometric response of the Ur-DSP/SPGE sensor versus time in the 0–600 μM NH_4_HCO_3_ range.

We confirmed the previously observed behavior by measuring the potential variation upon successive addition of an NH_4_HCO_3_ standard solution (Figure 4c). Worthy of note, we obtained a Δ*V* of 170 mV upon 600 μM urea. Our measurements reported in Figure 4a show an Δ*V* of 176 mV upon 600 μM urea. This final observation evidences a quantitative urea hydrolysis catalyzed by urease, the enzyme in our Ur-DSP/SPGE.

The potentiometric response is affected by various parameters, e.g., concentration and pH of the buffer, loading of the enzyme, and temperature.^56^ Therefore, we evaluated the optimal working pH of the new biosensor, and the maximum potential change (Δ*V*) was observed at the physiological pH = 7.4 (Figure S2a) suited for urease to activate the enzymatic decomposition of urea.^11,57^

In addition, we investigated the influence of the amount of DSP during the sensor fabrication by varying its deposited volume from 1 to 15 μL while keeping constant the urease amount (10 UI). Figure S2b shows that Ur-DSP/SPGE fabricated by depositing 10 μL of DSP gives a larger Δ*V* upon the addition of 200 μM urea, probably because of a total electrode surface coverage with a DSP monolayer. Lower surface coverage results in a lower signal, while higher DSP quantity could result in a DSP multilayer that partially hinders the electrical communication between electrode surface and solution. Figure S2c shows the potentiometric response of Ur-DSP/SPGE fabricated using 1–20 UI urease, upon the addition of 200 μM urea. Once more, urease concentrations lower than 10 UI probably do not result in a urease monolayer on the DSP-functionalized SPGE, thus giving lower potential variations. Higher urease concentrations, on the other hand, provide a potential change comparable to that of the sensor synthesized using 10 UI of urease. As a consequence, the optimal conditions for the Ur-DSP/SPGE sensor were represented by pH of 7.4, 10 μL of DSP, and 10 UI of urease.

Also Figure S3 shows the dynamic response obtained from the Ur-DSP/SPGE sensor after the addition of 200 μM urea to the 0.01 M PBS solution. Initially, the registered potential rapidly decreases, and then it gradually goes toward stabilization, remaining almost constant after around 300 s (response time).

Figure 5 shows the OCP curves for the bare SPGE (a) and Ur-DSP/SPGE (b) immersed in 0.01 M PBS solutions also with 0–600 μM urea concentrations. Figure 5c shows the calibration curve for Ur-DSP/SPGE.

**Figure 5:**
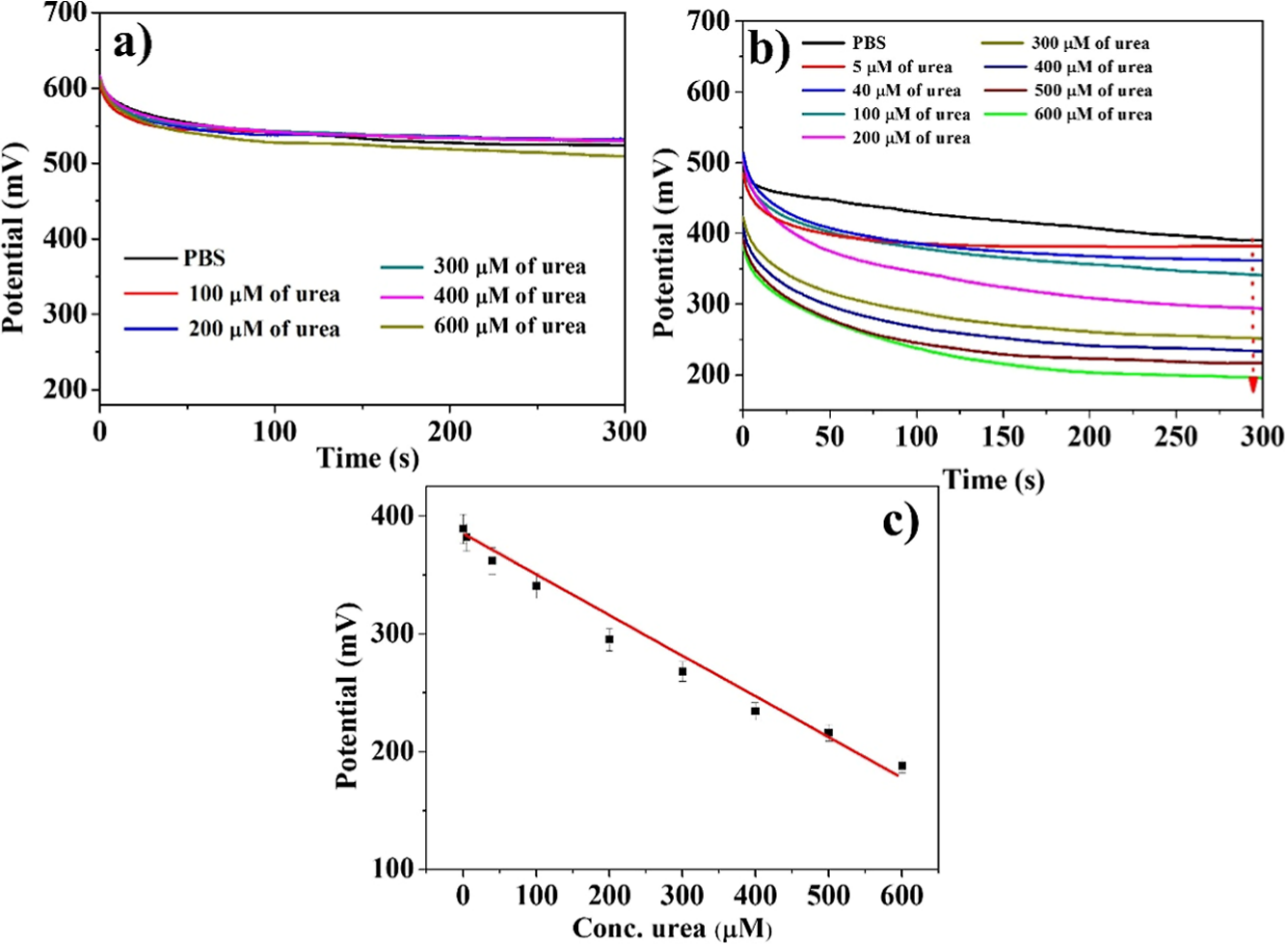
OCP response upon 0–600 μM urea using (a) SPGE and (b) Ur-DSP/SPGE. (c) Calibration curve for Ur-DSP/SPGE (*y* = *a* + *bx*; *R* ^2^ = 0.9997; intercept = 388.66667 ± 0.74536; slope = −0.47 ± 0.00577);.

The potential change of the Ur-DSP/SPGE sensor registered at the highest tested urea concentration (600 μM) was approximately 200 mV, which is very high compared to that observed under the same conditions with the bare SPGE (<30 mV). This potential change also showed a linear trend vs the urea concentration. The obtained LOD was 5.0 μM.

The repeatability of the Ur-DSP/SPGE sensor was studied through repeated OCP analyses using the same biosensor and alternating immersion in a 200 μM urea solution with washing in distilled water. Results obtained over six cycles are strongly indicative of a good repeatability with an RSD of 2.6% (Figure 6a). The stability overtime for Ur-DSP/SPGE was confirmed by testing the same sensor at 0, 30, 60, and 90 days after its preparation since the sensor maintains a good performance up to 60 days (RSD = 4.2%, Figure 6b).

**Figure 6:**
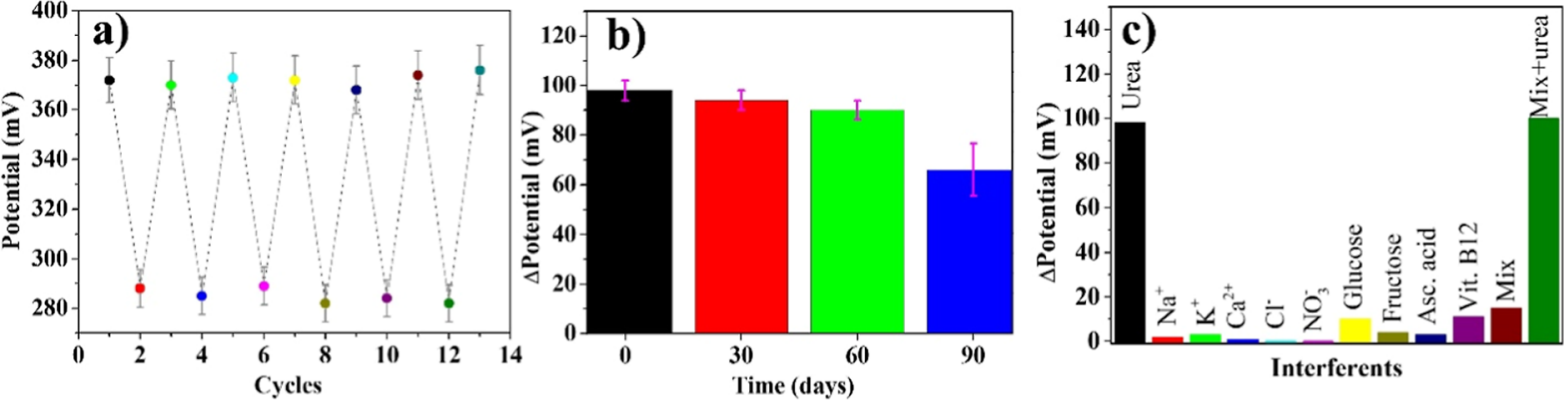
(a) OCP measurements using the same Ur-DSP/SPGE sensor by cycling six times in the absence and presence of 200 μM urea; (b) OCP measurements using the same Ur-DSP/SPGE sensor upon the addition of 200 μM urea at different times; (c) OCP measurements using the same Ur-DSP/SPGE sensor upon the addition of 200 μM urea or 200 μM interferents or upon the addition of a mixture of all of them.

In addition, 5 freshly prepared Ur-DSP/SPGE sensors were used to measure 100 μM urea in 0.01 M PBS. All five electrodes showed similar OCP responses with an RSD of 1.68%, thus stressing the high reproducibility of Ur-DSP/SPGE (Figure S4).

The Ur-DSP/SPGE sensor was also tested in the presence of some interfering species such as Na^+^, K^+^, Ca^2+^, Cl^–^, NO_3_^–^ glucose, fructose, ascorbic acid, and vitamin B12. In all these cases, a relevant potential change was observed only when urea was present (Figure 6c), thus demonstrating a high sensor selectivity due to the monolayer of the highly specific enzyme covalently grafted on the sensor surface.^58^

### Real Sample Analysis

The Ur-DSP/SPGE sensor was also tested for real samples of saliva, cow’s milk, and tap water to verify its urea detecting ability in complex matrices. Measurements were made before and after the addition of 0.1 mL of each of the above given samples to 3.9 mL of the PBS water solution (1:40). Figure 7 shows a Δ*V* of 27 (a) and 33 mV (b) for saliva and milk, respectively. From the calibration line (Figure 5c), urea amounts of 54.0 and 67.0 μM were evaluated, and considering the 1:40 dilution, we found concentrations of 2.1 and 2.6 mM in saliva and milk, respectively. These values are strongly in agreement with those expected for physiological conditions. The goodness of our Ur-DSP/SPGE sensor was also validated by urea sensing using the NADH method (see the calibration curve obtained with Urea Assay Kit III in Figure S5).

**Figure 7:**
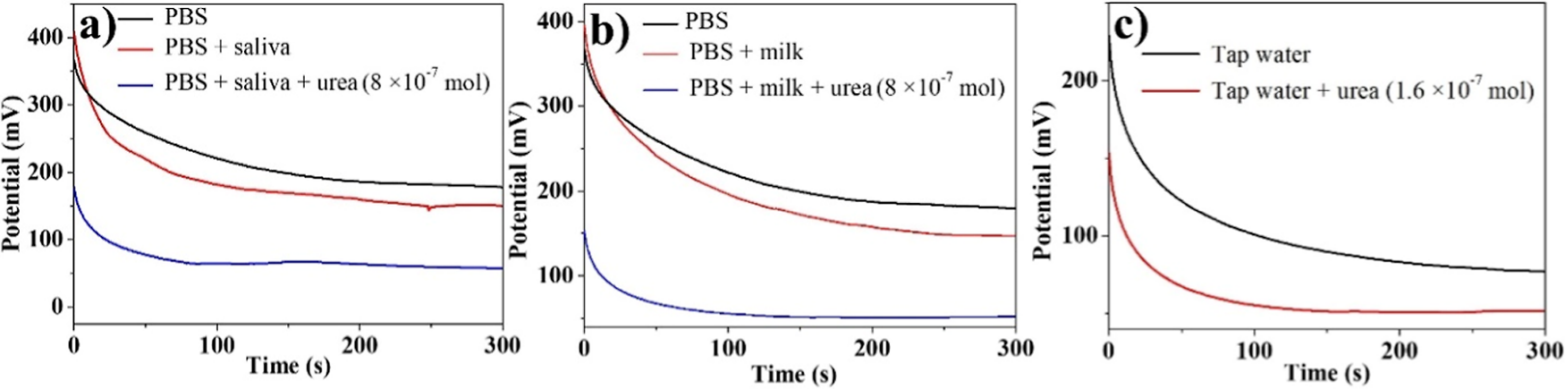
OCP analyses using the Ur-DSP/SPGE sensor for the determination of urea in (a) saliva, (b) milk, and (c) tap water.

The further addition of 80 μL of a 10 mM urea solution (8 × 10^–7^ mol, 200 μM) to 0.1 mL of saliva or milk plus 3.9 mL of the PBS water solution resulted in 0.201 (for saliva) and 0.203 (for milk) mM urea final solutions (total volume = 4.08 mL) and the measured potential variation allowed us to evaluate the associated Recovery (Figure 7, Table 1). Also, measurements performed for urea free tap water, after the addition of urea to obtain a 40.0 μM urea solution (Figure 7c), demonstrated the excellent ability of Ur-DSP/SPGE to detect traces of urea in potable water. In all cases, the sensor’s ability to detect urea is evident, with recoveries ranging between 95 and 99%, and these measurements further stress the possibility to detect urea in real samples.

**Table 1:** Ur-DSP/SPGE Sensor Response to Urea in Real Samples^a^

sample	added urea (40 μM)/measured urea (μM)	total urea (μM)/measured urea (μM)	recovery (%)	RSD (%)
PBS 0.01M	40/40	200/200	100	0.9
saliva		201/190	94.5	2.1
milk		203/195	96.1	2.6
water	40/39.4		98.5	1.5

a Estimated uncertainties 0.1 mV.

Our Ur-DSP/SPGE sensor shows a LOD of 5 μM, which is the state-of-the-art, better than many other already reported enzymatic and nonenzymatic sensors, as shown in Table 2. It is important to note that our sensor does not require complex sensor synthesis, can be used by simply immersion in the medium to be analyzed, and can be restored by simple washing in water.

**Table 2:** Comparison of the Urea Detection Performance of Ur-DSP/SPGE and of Some Reported Sensing Systems

sensors	linear range (mM)	LOD (μM)	method of detection	references
NiO/cellulose/CNT	0.01–1.4	7	nonenzymatic	^59^
Ni@NiMn	1–9	55.6	nonenzymatic	^60^
Electr.sens integrated tip-like sensor	0.5–7.0	20	nonenzymatic	^61^
urea-PEDOT/C–Au NTs EC sensor	1–100	100	enzymatic	^62^
La–CoFe LDH@rGO	0.001–23.5	0.33	nonenzymatic	^63^
MNA-MC	3–18	900	enzymatic	^64^
PVdF/Ni–Co(0.5:0.5)	0.02–2.0	12	nonenzymatic	^65^
Ur-DSP/SPGE	0.005–0.6	5	enzymatic	this work

In summary, screen-printed gold electrodes allowed an easy fabrication of a biosensor by covalently anchoring the urease enzyme on a gold working electrode, using for the first time the DSP bifunctional linker as a coupling agent. This system has led to a new biosensor with excellent capabilities for selective urea monitoring (μM) in laboratory samples and real milk, saliva, and tap water. The biosensor was well characterized by CV, EIS, and FTIR and demonstrated good sensitivity, reversibility, and long-term stability (about 60 days). The synthesis method for Ur-DSP/SPGE proved to be highly reproducible. Open circuit potentiometry allowed the measurement of the optimal limit of detection for urea. The performance of our Ur-DSP/SPGE sensor was also supported by urea sensing using the NADH method. The best pH value for urea sensing was around 7 since urea degradation involves the formation of bicarbonate and ammonium ions. From this study, it emerges that the use of DSP paves the way for the fabrication of new selective, sensible, reversible, and robust biosensors, and the present developed biosensor can be useful for health, food fraud control, and environmental pollution applications.

## Supplementary Material



## Notes

Assignation of FTIR bands; Ur-DSP/SPGE sensor response for a 500 μM urea solution subsequently diluted with 40 and additional 40 μL of PBS solution; OCP analysis, measured 3 times (RSD ≤ 2.6%); OCP for Ur-DSP/SPGE upon 200 μM urea at different pH values (5.0, 6.0, 7.4, 8.0 and 9.0), amount of DSP linker (1, 5, 10, and 15 μL of a 10 mg/mL stock solution, 2.47 × 10^–5^ mol/L), and urease concentration (2, 5, 10,15 and 20 UI); OCP response versus time of Ur-DSP/SPGE to 200 μM of urea; OCP response toward 100 μM urea for five Ur-DSP/SPGE; and calibration line obtained by UV–vis measurements at 340 nm, using a Urea Assay Kit III (Sigma-Aldrich) for the detection of NADH (PDF)
