# Supplementary material for: Biosensing of Urea with a Functionalized Gold Electrode for Health and Food Monitoring
Source: J Agric Food Chem. 2025 Sep 24;73(40):25628–35. doi: 10.1021/acs.jafc.5c08426 (PMC12512190; doi:10.1021/acs.jafc.5c08426)
Supplement: Supplementary file 1 [file jf5c08426_si_001.pdf]

Supporting information

Biosensing of Urea with a Functionalised Gold Electrode for health and food monitoring

Angelo Ferlazzo†, Meryam Chelly‡*, *Antonino Gulino†* and *Giovanni Neri‡*

†Department of Chemical Sciences and INSTM Research Unit, University of Catania, Viale Andrea Doria 6, 95125 Catania, Italy. (angelo.ferlazzo@unict.it)

‡Department of Engineering, University of Messina, Contrada Di Dio, 98166 Messina, Italy

Table S1. Assignation of FTIR bands of the SPGE (a), DSP/SPGE (b), Urease (c) and Ur-DSP/SPG (d).

Wave number (cm^{-1})				Peak assignments
**A**	**B**	**C**	**D**	
3420	3400			O-H stretching of water on the electrode surface.
			3252	O-H stretching and N-H stretching of primary and secondary amines and amides of proteins
		3000-3100		O-H and N-H stretchings for the proteins
	1712			-C = O stretching of carboxylic acids
		1648		-C=O peptide stretching
			1640	-C=O amide stretching
			1568	N-H bending
			1396	-C-H bending of alkanes
			1244	C-O stretching of alcohols, carboxylic acids, esters, ethers
		1140		C-O of carboxylic acid
			1064	C-N stretching for primary and secondary amines
	1020			N-C-O group
			980	C-N stretching
			856	N-H wag

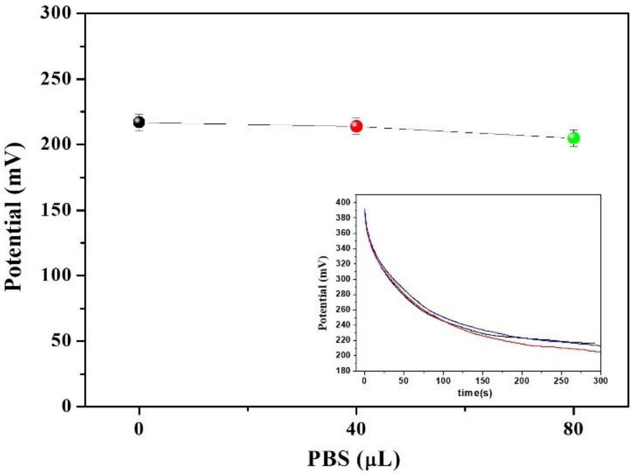

Figure S1. Ur-DSP/SPGE sensor response for a 500 μM urea solution (black sphere) subsequently diluted with 40 (line sphere) and additional 40 μL of PBS solution (line sphere); insert: OCP analysis, measured 3 times (RSD ≤ 2.6%).

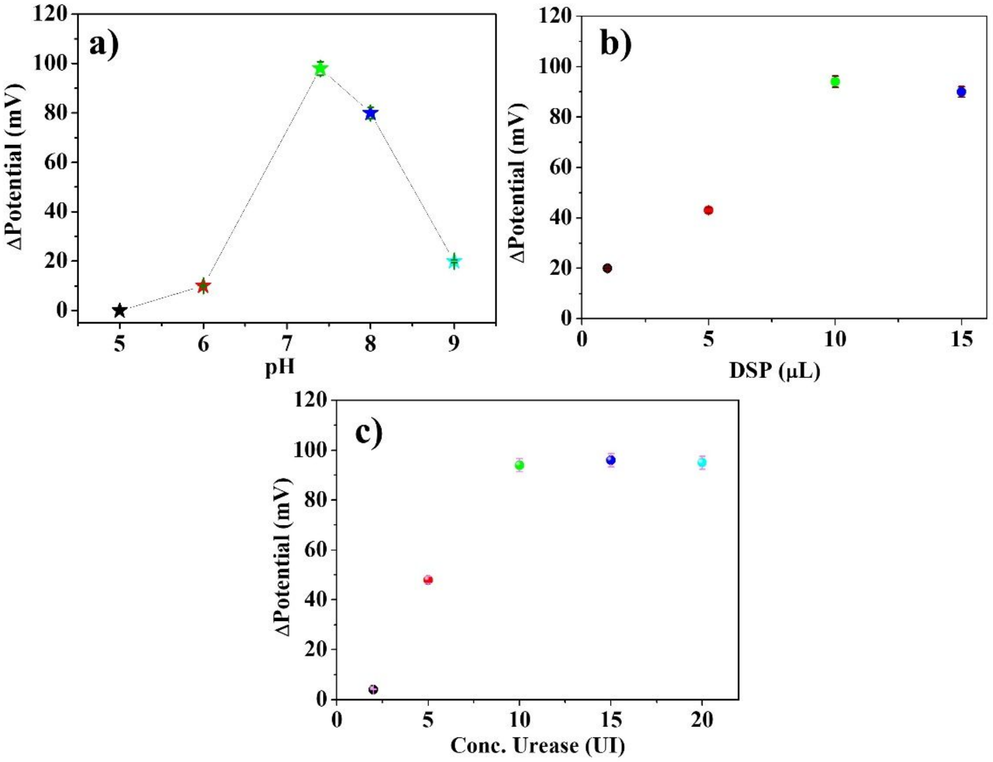

Figure S2. OCP for Ur-DSP/SPGE upon 200 μM urea at different: a) pH values (5.0, 6.0, 7.4, 8.0 and 9.0); b) amount of DSP linker (1, 5, 10 and 15 μL of a 10 mg/mL stock solution, 2.47×10^{-5} mol/L); c) urease concentration (2, 5, 10,15 and 20 UI); All measurements were repeated 3 times. (RSD ≤ 2.8%).

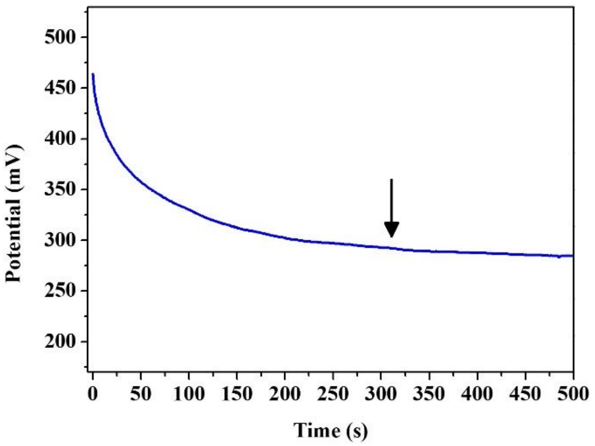

Figure S3. OCP response versus time of Ur-DSP/SPGE to 200 μM of urea

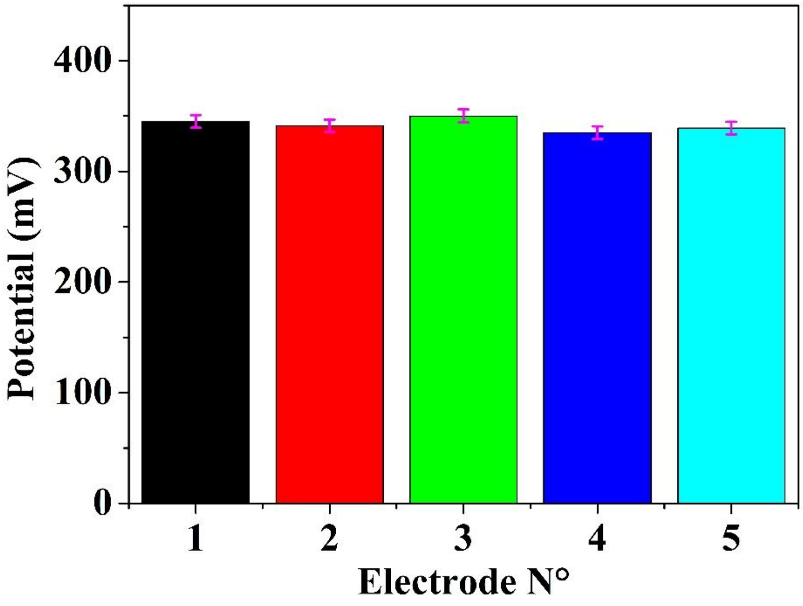

Figure S4. OCP response toward 100 μM urea for five Ur-DSP/SPGE.

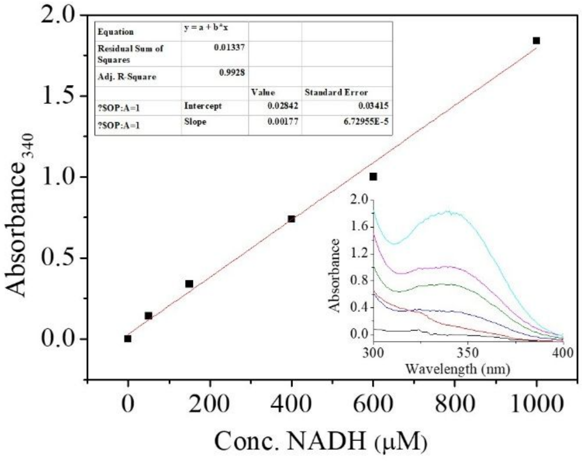

Figure S5. Calibration line obtained by UV-vis measurements at 340 nm, using a Urea Assay Kit III (Sigma-Aldrich) for the detection of NADH; inset UV-Vis spectra.
